# The Chain-Mediation Pathway of Social Avoidance to Depression in College Students Is Regulated by Self-Esteem

**DOI:** 10.3389/fpsyg.2022.802161

**Published:** 2022-05-17

**Authors:** Ye Yuan, Suhua Jiang, Xi Wen, Zhong Han, Daili Wu, Xuanping Wang, Tingyang Ye, Yimin Hu, Jaesik Jeong, Min Xiang

**Affiliations:** ^1^School of Mental Health, Wenzhou Medical University, Wenzhou, China; ^2^School of Mathematics and Statistics, Shangqiu Normal University, Shangqiu, China; ^3^School of Public Health and Management, Wenzhou Medical University, Wenzhou, China; ^4^Department of Statistics, Chonnam National University, Gwangju, South Korea; ^5^School of Stomatology, Wenzhou Medical University, Wenzhou, China

**Keywords:** social avoidance, interpersonal trust, loneliness, self-esteem, depression

## Abstract

**Objective:**

Here, we investigated the relationship between social avoidance and depression in college students, explored the mediating roles of loneliness and trust, and the regulatory role of self-esteem, to provide a theoretical intervention approach based on internal mechanisms.

**Methods:**

Using a simple random overall sampling method, 1,021 college students were investigated using self-rating depression, social avoidance and distress, loneliness, interpersonal trust and self-esteem scales.

**Results:**

There was a significant positive correlation between social avoidance and depression. Loneliness and interpersonal trust played chain-mediating roles between social avoidance and depression. The influence of social avoidance on interpersonal trust was regulated by self-esteem. Specifically, the social avoidance level of the low self-esteem group was more likely to be affected by interpersonal trust issues.

**Conclusion:**

Social avoidance not only directly affects college students’ depression, it also has indirect effects through interpersonal trust and loneliness. Thus, interpersonal trust and loneliness have chain-mediating effects between social avoidance and depression in college students, and self-esteem regulates the mediation process.

## Introduction

Depression is an abnormal depressed and unpleasant negative emotional state that has a negative impact on personal lives and society (Qian et al., 2019). It is a global clinical and social problem. There is a high incidence of depression among college students, and it is presently their main mental health problem (Xu, 2010). Depression has become an invisible negative influence on college students’ psychology. College students are the future societal pillars; consequently, it is important to explore the influencing factors behind depression and its mechanisms to provide more effective prevention and intervention strategies. The psychological stress-process model based on Unified Modeling Language (UML) modeling divides the stress process into several stages, such as stressors, stress responses, coping, and results. The change from one stage to the next actually reflects a change in the system state. Stress response refers to the physiological and psychological reactions and behavioral changes caused by stressors, indicating an individual’s adaptation to changing internal and external environments (Jiang, 2012). Coping has been described as an individual’s constantly changing cognitive and behavioral efforts to deal with specific internal and external environmental requirements that are evaluated by themselves as beyond the scope of their own abilities and resources (Yu, 1994). And human depression is also often a stress response to social avoidance. Social avoidance and distress are the avoidance behaviors and feelings of individuals during social interactions, and they are specifically manifested as negative emotions and associated withdrawal and escape behaviors (Zhang et al., 2018). Therefore, social avoidance may be regarded as a stress response, whereas depression may be regarded as the result of stress. Loneliness is an emotional experience. It includes emotional and social isolation and the emotional experience of emptiness, anxiety, and unease caused by an individuals’ dissatisfaction with the quantity and quality of current interpersonal relationships (Tu and Zhang, 2015). Loneliness stems from unmet intimacy needs and a lack of early attachment in childhood (Weiner, 1975). It is a subjective state of mind, and people may feel lonely, even if they are not socially isolated (Heinrich and Gullone, 2006). Social avoidance and distress have significant positive predictive effects on loneliness (Dong, 2020). Interpersonal trust refers to an individual’s generalized expectation that the words, promises, and oral or written expressions of another person or group are reliable (Rotter, 1967). Lother and Letterman regard trust as a personality characteristic or a belief that exists within the individual. Interpersonal trust reflects an individual’s trust in other people’s words and deeds, as well as motivations and personalities (Xu and Pang, 2004). Yang and Peng (1999) believe that interpersonal trust is a sense of guarantee that both parties are able to fulfill their entrusted obligations and responsibilities. Therefore, in the face of stress, changes in people’s trust level and their loneliness may be regarded as coping mechanisms. There is a correlation between social avoidance and trust. The greater the avoidance of, and distress during, social activities, the lower the level of trust in others (Yi, 2016).

A significant correlation exists between social avoidance and depression, but the internal mechanisms of social avoidance and depression in college students have been rarely explored. Additionally, from the environment–individual interaction model (Kumpfer, 2002), it can be inferred that social avoidance not only has a direct effect on depression, it also affects the level of depression through interactions with its own factors. Loneliness and interpersonal trust may be two important individual factors. There is a significant negative correlation between social avoidance and interpersonal trust (Wang, 2009) and Jin and others found that there is a positive correlation between social avoidance and loneliness (Jin et al., 2014). Additionally, the higher the loneliness score, the higher the depression score (Dai et al., 2017) and Martínez et al. (2019) found that there is a significant correlation between interpersonal trust and depression.

Self-esteem is an individual’s evaluation and experience of self-worth, and its core components include self-competence and self-acceptance (Tafarodi and Swann, 1995). Self-esteem can regulate the relationship between perceived social support and social avoidance (Li et al., 2016). Thus, with an improved level of perceived social support, the social avoidance and distress of individuals with high self-esteem decreases more rapidly.

Although previous studies have established a single relationship between loneliness and depression, social avoidance and depression, and social avoidance and interpersonal trust, However, it is not clear whether self-esteem plays a regulatory role between social avoidance and interpersonal trust. the relationship between self-esteem and interpersonal trust, loneliness, social avoidance and depression is diverse and complex. At present, there is no research report, in which there is a clear direct correlation.

In summary, this study constructed a chain-mediation effect model in accordance with the psychological stress-process (Jiang, 2012) and the environment–individual interaction (Kumpfer, 2002) models, and it comprehensively examined the mechanisms between social avoidance and depression in college students to explore the mediating roles of interpersonal trust and loneliness in this process, as well as the regulatory role of self-esteem.

Based on this, the following hypotheses are put forward: (1) it has a positive predictive effect on College Students’ depression; (2) interpersonal trust and loneliness play independent mediating roles between social avoidance and depression; (3) interpersonal trust and loneliness play a chain intermediary role between social avoidance and college students’ depression, that is, “social avoidance → interpersonal trust → loneliness → depression.”

## Materials and Methods

### Participants

With a convenient sampling method, we recruited 1,050 college students (397 come from WenZhou Medical University, 560 come from WenZhou University, and 64 come from Wenzhou Polytechnic University). We collected 1,050 questionnaires through our survey, and 1,021 were valid (including 570 females), yielding a valid response rate of 97.23%. All participants were 18–24 years old. There were 374 freshmen, 429 sophomores, 141 juniors, and 77 seniors. Among them, 67.5% majored in Science and Engineering, 29.4% majored in Medicine, 1.7% majored in Literature and History, and 1.3% majored in Art. Additionally, 63.3% were registered rural residents and 45.6% were only children.

### Measures

#### Self-Rating Depression Scale

There were 20 questions subjected to the self-rating depression scale (SDS) compiled by Zung. They were used to evaluate the subjective feelings of the subjects over the last week, and were related to four factors: psychotic emotional symptoms, somatic disorders, psychomotor disorders, and psychological disorders related to depression. A four-level scoring system was used, in which “1” represented no or little of the time, “2” represented a small part of the time, “3” represented a considerable amount of the time, and “4” represented most or all of the time. The higher the score, the more obvious the depressive tendency. The α-coefficient in this study was 0.890, which indicated good reliability and valid construction.

#### Social Avoidance and Distress Scale

The social avoidance and distress scale (SAD), compiled by Watson and others, contains 28 questions, of which 14 are used to evaluate social avoidance and 14 are used to evaluate social distress, with the total score focusing on aspects of social avoidance. The initial scoring method was “Yes–No”; therefore, the scores ranged from 0 to 28. The SAD definition of social avoidance does not mean that one cannot participate in social interactions. The higher the score, the greater the social avoidance tendency. The α-coefficient in this study was 0.79, which indicated high reliability and validity.

#### Loneliness Scale

The Loneliness Scale (UCLA), compiled by Russell, is used to evaluate loneliness caused by the gap between the desire for social communication and the actual level, which is defined as one-dimensional. There were 20 questions, with answers scored using a four-level system, in which “1” represented I have never felt this way, “2” represented I rarely feel this way, “3” represented I sometimes feel this way, and “4” represented I often feel this way. Nine of the questions were scored in reverse, and the higher the score, the more obvious the loneliness tendency. The α-coefficient in this study was 0.906, which indicated high reliability and validity.

#### Interpersonal Trust Scale

For the interpersonal trust scale (IT), compiled by Rotter, there were 25 questions, which were scored using a five-point symmetrical system, with “1” representing complete agreement and “5” representing complete disagreement. The total scores ranged from 25 to 125. The scale is used to test the subjects’ estimation of the reliability of other people’s behaviors, commitments, or statements, and the higher the score, the higher the degree of trust. The α-coefficient in this study was 0.755.

#### Self-Esteem Scale

In the self-esteem scale (SES), compiled by Rosenberg, there are 10 questions. The answers were scored using a four-level method, in which “1” indicated very consistent, “2” indicated compliance, “3” indicated non-conformity, and “4” indicated very non-conforming. The scale is used to assess teenagers’ overall feelings of self-worth and self-acceptance, and the higher the score, the higher the level of self-esteem. The α-coefficient in this study was 0.844, which indicated high reliability and validity.

### Procedure and Statistical Analysis

In accordance with the Helsinki Declaration, this study was con-:ducted after approval from the ethics committees of WenZhou Medical University. Before participation, everyone either provided their oral consent or checked a box indicating informed consent on the online questionnaires. Participants were given instructions regarding each scale before rating the items. They were invited to fill out the five self-report questionnaires in sequence anonymously and voluntarily, and they were free to terminate the participation at any time.

SPSS17.0 was used for descriptive statistical analysis and correlation analysis. Mplus 7.0 was used for structural equation model analysis.

## Results

### Control of Common Method Deviation

Because the data in the study were derived from self-evaluations by the research subjects, the common method deviation (Tang and Wen, 2020) effect may be present. In accordance with Podsakoff’s suggestion, this study strictly controlled the testing procedure: all the questionnaires were anonymous and the scoring scales were all widely used with high reliability and validity. The Harman single factor test was selected to diagnose the common method deviation. The variance explained by the first factor was 19.54%, which was less than the critical value of 40%. Therefore, there was no common method deviation in the study data.

### Descriptive Statistics and Correlation Analysis of Each Variable

As shown in Table 1, there was a significant correlation between depression and social avoidance, as well as significant positive correlations between depression and interpersonal trust, loneliness, and self-esteem. Significant negative correlations existed between social avoidance and both loneliness and interpersonal trust, but social avoidance did not significantly correlate with self-esteem. There were significant positive correlations between loneliness and both interpersonal trust and self-esteem, and there was a significant positive correlation between interpersonal trust and self-esteem.

**TABLE 1:** Descriptive statistics and correlations among variables.

Variable	−x ± s	SDS	SAD	UCLA	IT
SDS	36.63 ± 9.05				
SAD	15.93 ± 7.35	0.583**			
UCLA	41.03 ± 9.75	0.671**	0.491**		
IT	75.48 ± 9.10	–0.437**	–0.410**	–0.493**	
SES	20.47 ± 4.60	0.687**	0.387**	0.613**	–0.399**

**P < 0.01. SDS, self-rating depression scale; SAD, social avoidance and distress scale; UCLA, loneliness scale, IT, interpersonal trust scale, SES, self-esteem scale.

### Mediating Effect Test

On the basis of the predictive effect of social avoidance on college students’ depression, combined with two important variables, interpersonal trust and loneliness, which are significantly related to depression, this study analyzed the path to depression and discusses the internal mechanisms behind the effects of social avoidance on depression.

**FIGURE 1:**
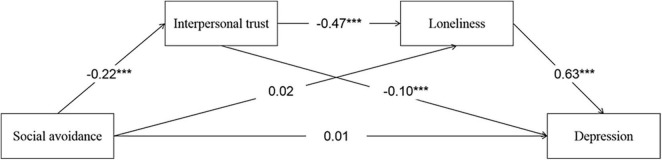
Chain-mediation pathway diagram. ***P < 0.001.

All the variables were standardized. The deviation correction method, with 5,000 bootstraps, was used to obtain a 95% confidence interval to test the significance of the effects. If the confidence interval does not contain 0, then the statistical result is significant. As shown in Figure 1, social avoidance had significant predictive effects on interpersonal trust (β = –0.22, SE = 0.03, *t* = –7.19, *P* < 0.001, 95%CI = −0.18∼−0.08,η^2^ = 0.21), loneliness (β = 0.02, SE = 0.03, *t* = 0.77, *P* < 0.001, 95%CI = 0.04∼0.15,η^2^ = 0.15), and depression (β = 0.01, SE = 0.03, *t* = 0.57, *P* < 0.001, 95%CI = 0.23∼0.36,η^2^ = 0.23), interpersonal trust had significant predictive effects on loneliness (β = –0.47, SE = 0.03, *t* = –16.24, *P* < 0.001, 95%CI = −0.46∼−0.33,η^2^ = 0.58) and depression (β = –0.01, SE = 0.03, *t* = –3.59, *P* < 0.001, 95%CI = −0.437∼−0.288,η^2^ = 0.28), and loneliness had a significant predictive effect on depression (β = 0.63, SE = 0.03, *t* = 23.46, *P* < 0.001, 95%CI = 0.363∼0.496,η^2^ = 0.19). The mediating effects, direct effects, and corresponding effect scales are shown in Table 2, and they indicated that loneliness and interpersonal trust play intermediary roles between social avoidance and depression.

**TABLE 2:** The 95% confidence interval of the mediating effect test and deviation corrections.

Effect	Path	Effect quantity	Effect quality	95%
Direct effect	Social Avoidance → Depression	–0.014	12.17%	[–0.06, 0.03]
Mediating effect	Social Avoidance → Interpersonal Trust → Depression	–0.022	19.13%	[–0.04, –0.01]
	Social Avoidance → Loneliness → Depression	–0.014	12.17%	[–0.05, –0.02]
	Social Avoidance → Interpersonal Trust → Loneliness → Depression	–0.065	56.52%	[–0.09, –0.05]
Total indirect effect	/	–0.101	87.83%	[–0.14, –0.06]
Total effect	/	–0.115	100%	[–0.18, –0.06]

### Mediating Effects of Regulation

As shown in Table 1, there was a significant correlation between self-esteem and interpersonal trust, and there was also a significant correlation between social avoidance and interpersonal trust. To determine whether self-esteem regulates interpersonal trust as an intermediary between social avoidance and depression, the pathway between social avoidance and interpersonal trust, regulated by self-esteem, was further tested. Social avoidance had a significant predictive effect on interpersonal trust (β = –0.20, SE = 0.03, *t* = –6.96, *P* < 0.001,η^2^ = 0.21), whereas self-esteem had a significant predictive effect on interpersonal trust (β = 0.34, SE = 0.03, *t* = 10.20, *P* < 0.001,η^2^ = 0.22). Additionally, the interaction between social avoidance and self-esteem had a significant negative predictive effect (β = 0.08, SE = 0.03, *t* = 2.11, *P* = 0.04,η^2^ = 0.39). This indicated that the predictive effect of social avoidance on interpersonal trust was regulated by self-esteem.

**FIGURE 2:**
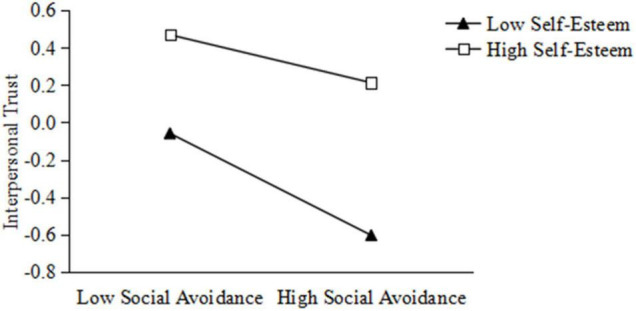
The regulatory effects of self-esteem on the relationship between social avoidance and interpersonal trust.

To understand the essence of the regulatory effect, the bootstrap method was used to verify the regulatory effect. The high and low self-esteem group scores were divided by the average value of self-esteem plus or minus a standard deviation. The mediating effect of the high self-esteem group was significant, and the total indirect effect value of the high self-esteem group was 0.06 (*P* = 0.004). The mediating effect of the low self-esteem group was more significant, and the total indirect effect value of the low self-esteem group was 0.12 (*P* < 0.001). There was a significant difference in the mediating effects between the two groups (*P* = 0.03). A simple slope analysis is shown in Figure 2. For the high self-esteem group, social avoidance had a significant negative predictive effect on interpersonal trust (β = –0.13, *t* = –3.00, *P* = 0.003), whereas for the low self-esteem group, social avoidance was a stronger predictor of interpersonal trust.

## Discussion

On the basis of the social motivation theory, this study used college students as the research subjects and investigated the relationships between social avoidance and loneliness, depression, and interpersonal trust, as well as the underlying mechanisms and the regulatory effects of self-esteem.

Descriptive discussion analysis found that, Descriptive statistics showed that depression was positively correlated with loneliness, interpersonal trust and self-esteem, but negatively correlated with social avoidance. Social avoidance has a significant positive correlation with loneliness and self-esteem, and a significant negative correlation with interpersonal trust. Loneliness has a negative correlation with interpersonal trust and a significant positive correlation with self-esteem. Interpersonal trust and self-esteem showed a significant negative correlation, and there is a significant correlation between each research variable.

A mediation effect analysis indicated a significant positive correlation between social avoidance and depression in college students, and the former had a positive predictive effect on the latter, which is consistent with the results of a previous study (Huang et al., 2019). Kompf’s environment–individual interaction model (Cohen and Wills, 1985) reveals that the intervention of stress or challenge successfully activates the risk and protective factors in the external environment, disrupting the individual’s internal stability. The emergence of social avoidance also disrupts the internal mental homeostasis of the body. The main effect model of social support (Cohen and Wills, 1985) reveals that social support has beneficial effects on the maintenance of an individual’s emotional, physical, and mental states during stressful situations. However, college students are in a period of prominent inconsistent psychology, and they lack the adaptability and psychological defense mechanisms to cope with setbacks (Li, 2009). Under various pressures, college students are prone to social avoidance, resulting in depression and other changes in their emotional state. The buffer model of social support (Cohen and Wills, 1985) states that social support can buffer the negative effects of setbacks on an individual’s body and mind, and a high degree of social support is a predictor of greater adaptability and fewer emotional problems. Thus, individuals experiencing social avoidance are less adaptable and are prone to emotional problems.

The mediating effects analysis revealed that interpersonal trust has a mediating effect on the relationship between social avoidance and depression. Interpersonal trust is a kind of psychological expectation or commitment–trust relationship between people (Xu, 2010). Lopez and Brennan (2000) found that adult attachment types can significantly affect the social cognitive processes of individuals. Compared with unsafe attachment individuals, secure attachment individuals show more accurate and highly differentiated evaluations of others, more positive and accurate attributions of peer behavior, more positive expectations of peers, and more trust in others. Our experimental results support the above conclusion that individuals with a high level of social avoidance rely less on other individuals, resulting in lower expectations and trust in others. A negative correlation between interpersonal trust and depression has been documented (Xu, 2010).

The mediating effects analysis revealed that loneliness also has a mediating effect on the relationship between social avoidance and depression in college students. Loneliness is related to dissatisfaction, loneliness, and unhappiness in social relations, which is consistent with previous research results (Li, 2003). Individuals who avoid social activities often do not get sufficient social support, which leads to a higher degree of loneliness (Xu and Yang, 2017). If short-term loneliness is not alleviated or eliminated, then it is easy to form long-term loneliness. There is a significant positive correlation between loneliness and depression in college students (Dai et al., 2017). College students affected by long-term loneliness are more likely to be depressed and unwilling to communicate with others (Guo et al., 2011). If this proceeds for a long time without a proper outlet, resulting in the situation not improving, then these college students are more likely to suffer from depression. Dai et al. (2017) found that the correlation between predicted depression and a state of loneliness was 25.3%, which could explain 6.4% of the variation in depression.

From the analysis of the above mediation results, it can be seen that the higher the social avoidance, the lower the individual’s trust in the outside world. Without external psychological support, it will enhance the individual’s sense of loneliness for a long time, and eventually lead to depression, which will deepen social avoidance and enter the cycle. Therefore, social avoidance affects one’s depression by affecting one’s interpersonal trust and loneliness.

Further analyses of the moderated mediation model revealed that the regulatory role of self-esteem on social avoidance influences the mediating effect of interpersonal trust on depression in the first half of the pathway, and in individuals with low self-esteem, social avoidance has a stronger negative predictive effect on interpersonal trust. Thus, self-esteem is an important protective mechanism for physical and mental health, and this is consistent with the results of a previous study (Fu et al., 2020). Individuals with low self-esteem do not experience the benefits of help and support from interpersonal relationships. Thus, showing more avoidance and rejection of interpersonal relationships easily produces self-negation (Orth et al., 2008) and forms negative emotions, making it difficult to establish and maintain good interpersonal trust with others (Gkika et al., 2018).

The study results provide important information that can be applied to related theories and preventive interventions against depression in college students. In theory, this study proposed that social avoidance reduces the level of depression through multiple pathways, and revealed two important factors related to college students’ depression: interpersonal trust and loneliness.

Research shows that we should pay more attention and support to students who avoid social interaction. We should try our best to reduce college students’ loneliness and increase students’ interpersonal trust, so as to reduce the possibility of depression. For example, in reducing college students’ loneliness. Owing to the characteristics of loneliness, it is suggested that schools provide corresponding group counseling methods to help students accept themselves and each other through social skills training, role-playing, and joint discussions. This will help them master communication skills and increase their communication-related confidence, so that they can obtain social support, especially emotional support, through communication with others in a group atmosphere (Li, 2003). In addition, individual counseling should be provided in cases when students do not believe that avoiding society and others will be disadvantageous, want to communicate but avoid or are not good at communication, or have many inner anxieties. Additionally, experts in medical institutions should also regularly organize special lectures on medical psychology in colleges and universities to introduce the emergence and treatment of depressive psychological diseases from a medical point of view, and they should communicate with psychological counselors in colleges and universities. Providing effective intervention to college students with psychological issues from many levels will allow them to cope with their problems rationally and well, so that both body and mind can develop (Coplan et al., 2010).

## Conclusion

This study proposes a hypothetical model of cyclic relationship that depression can bring certainty to self. The model is helpful to formulate targeted intervention programs. This study had the following shortcomings. First, the data came from subjective self-rating reports, which may result in deviations. Future research needs a combination of other evaluations and self-evaluations to increase the objectivity. Second, there are many factors that cause depression among college students, and other psychological factors, such as introversion and extroversion, should be controlled for in the future. Third, as a cross-sectional correlation study, this study cannot determine the causal relationship, and subsequent experimental research is needed to further verify the causal hypothesis model. Finally, there may be mechanisms involving other variables between social avoidance and depression. Additionally, it is also worth exploring the factors that social avoidance influences in the second half of the interpersonal trust-related mediation pathway of depression. Clarifying the internal mechanisms between social avoidance and depression will aid in the development of improved intervention strategies.

## Data Availability Statement

The raw data supporting the conclusions of this article will be made available by the authors, without undue reservation.

## Author Contributions

MX and JJ made great contributions to the conception, design, data acquisition, manuscript writing, and modification of this study. YY organized the database and conducted the statistical analysis. XW, ZH, DW, XPW, YH, and TY were responsible for collecting and pretreating the data. SJ wrote the first draft of the manuscript. All authors were involved in the revision and reading of the manuscript and approved the submitted version.

## Conflict of Interest

The authors declare that the research was conducted in the absence of any commercial or financial relationships that could be construed as a potential conflict of interest.

## Publisher’s Note

All claims expressed in this article are solely those of the authors and do not necessarily represent those of their affiliated organizations, or those of the publisher, the editors and the reviewers. Any product that may be evaluated in this article, or claim that may be made by its manufacturer, is not guaranteed or endorsed by the publisher.
